# Inflammation as the nexus: exploring the link between acute myocardial infarction and chronic obstructive pulmonary disease

**DOI:** 10.3389/fcvm.2024.1362564

**Published:** 2024-02-21

**Authors:** Eloise Marriott, Aran Singanayagam, Juma El-Awaisi

**Affiliations:** ^1^ Microcirculation Research Group, Institute of Cardiovascular Sciences, College of Medical and Dental Sciences, University of Birmingham, Birmingham, United Kingdom; ^2^ MRC Centre for Molecular Bacteriology & Infection, Department of Infectious Disease, Imperial College London, London, United Kingdom

**Keywords:** chronic obstructive pulmonary disease, acute myocardial infarction, inflammation, anti-inflammatory, interleukins, S100A8/A9

## Abstract

Chronic obstructive pulmonary disease (COPD), particularly following acute exacerbations (AE-COPD), significantly heightens the risks and mortality associated with acute myocardial infarction (AMI). The intersection of COPD and AMI is characterised by a considerable overlap in inflammatory mechanisms, which play a crucial role in the development of both conditions. Although extensive research has been conducted on individual inflammatory pathways in AMI and COPD, the understanding of thrombo-inflammatory crosstalk in comorbid settings remains limited. The effectiveness of various inflammatory components in reducing AMI infarct size or slowing COPD progression has shown promise, yet their efficacy in the context of comorbidity with COPD and AMI is not established. This review focuses on the critical importance of both local and systemic inflammation, highlighting it as a key pathophysiological connection between AMI and COPD/AE-COPD.

## Introduction

Chronic obstructive pulmonary disease (COPD) is the third leading cause of death worldwide, with cardiovascular diseases (CVDs) ultimately contributing for approximately 50% of these deaths. Individuals with COPD have a significantly higher risk of acute myocardial infarction (AMI) and increased mortality from AMI (1), *particularly* during the first month following an acute exacerbation (AE)-COPD event. Indeed, AE-COPD patients are at an eightfold increased risk of suffering from an AMI in the first 3 days following hospitalisation (2). This is largely due to inflammation, both local and systemic, which is a major pathophysiological mechanistic link between AE-COPD/COPD and AMI incidence/mortality. While the contribution of various inflammatory pathways has been studied separately in each of these vascular beds, there is a lack of understanding around the thrombo-inflammatory crosstalk between these organs in health and disease. In this review we will explore: (i) the inflammatory factors involved in the interactions in and between COPD/AE-COPD and AMI; (ii) anti-inflammatories used in COPD/AE-COPD and AMI separately; and (iii) the unmet clinical needs in the treatment of patients with COPD with AMI.

## Inflammatory mechanisms in acute myocardial infarction

**Figure 1:**
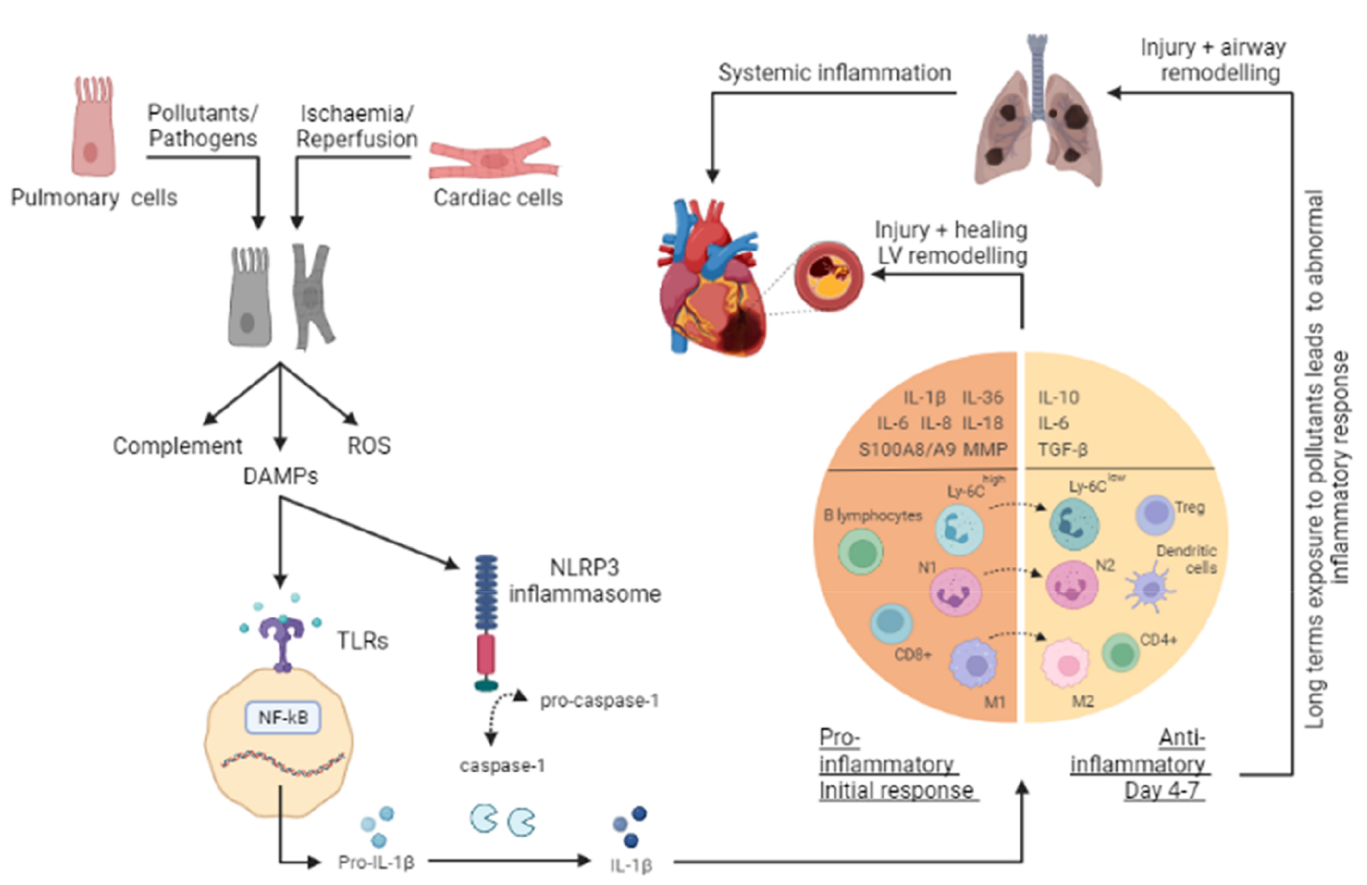
Overview of the inflammatory response in a COPD and AMI. The onset of either acute myocardial infarction (AMI) or chronic obstructive pulmonary disease (COPD) leads to cellular injury and necrosis of cardiac cells and pulmonary cells respectively, resulting in the release of intracellular content such as damage-associated molecular patterns (DAMPs), production of reactive oxygen species (ROS) and activation of the complement cascade. Once a DAMP (such as DNA, mitochondrial DNA (mtDNA), RNA, and the nuclear factor - high mobility group box 1 (HMGB1), and ATP) is recognised by toll-like receptors (TLRs), cytoplasmic myeloid differentiation factor 88 (MyD88) is activated. This in turn, stimulates the NF–κB pathway and through a signaling cascade and transcription, leads to the release of several inflammatory mediators such as pro-interleukin (IL)-1β. Additionally, in response to the release of DAMPs, inflammasomes (such as nucleotide-binding oligomerization domain receptor family protein 3, NLRP3) are activated to promote the cleavage of pro-Caspase-1 into Caspase-1, which in turn processes the pro-IL-1β into the active IL-1β form. As a result, pro-inflammatory mediators (IL-1β, IL-1α, IL-6, IL-8, IL-18, IL-36, S100A8/A9, and MMPs) and cell adhesion molecules are increased at the site of injury. Extravasation and recruitment of neutrophils and other leucocytes (monocytes, macrophages, B lymphocytes, and CD8+ T cells) to the site then occurs as a result of the interactions between endothelial cells and infiltrating leucocytes. The subsequent anti-inflammatory phase (day 4-7) facilitates the resolution and repair through anti-inflammatory cytokines (IL-10, IL-6, and TGF-β), changes in macrophages (M1 to M2) and monocytes (Ly6Chigh to Ly6Clow), and recruitment of cells (T-regs, dendritic cells, CD4+ T cells). This response serves to help wound healing; however, a prolonged and excessive inflammatory response can worsen these conditions, leading to further injury and remodeling. Additionally, long term exposure to pollutants can enhance and alter the inflammatory response in the lungs, resulting in a progressive and persistent inflammatory response.

The onset of an ischaemic event during an AMI, and reperfusion through primary percutaneous coronary intervention (PCI), can each induce inflammation because of cellular injury and death to cardiac myocytes, endothelial cells, and fibroblasts. This inflammation initiates through various processes, including the release of necrotic cell contents, reactive oxygen species (ROS) production, and activation of the complement system. Biomarkers such as extracellular DNA, mtDNA, RNA, and HMGB1, known to be elevated post-AMI, play dual roles in inflammation and cardioprotection, as indicated by the effectiveness of DNAase, RNAase1, or HMGB1 inhibitors in reducing MI severity (3, 4). The inflammatory cascade involves the production of chemokines, cytokines, and adhesion molecules at the infarct site (Table 1), followed by the recruitment of neutrophils and other leukocytes, aiding in wound healing and scar formation (22). However, a prolonged and excessive inflammatory response can worsen the condition, leading to larger infarct size, left ventricular (LV) remodeling, and heart failure (Figure 1). The inflammatory response following myocardial ischemia-reperfusion (IR) injury is mediated through the coordinated activity of several types of cells, both within and distant to the infarcted area. A hallmark among patients with AMI is leucocytosis which has been widely used as a predictor of mortality within this group of patients. Neutrophils are thought to be the first type of cells to be recruited during myocardial IR injury, followed by monocytes, mast cells, and lymphocytes. Each of these cells has a distinct role within the inflammatory process during myocardial IR injury (23, 24).

**Table 1:** Inflammatory mediators in chronic obstructive pulmonary disease and acute myocardial infarction.

Inflammatory mediator	Chronic obstructive pulmonary disease	Acute myocardial infarction	References
Targets which have undergone clinical trials			
IL-1	Promotes emphysema onset and airway remodeling. Upregulated in AE-COPD	Upregulates pro-inflammatory mediators, adhesion molecules and leucocyte recruitment.	(5–8)
IL-6	Promotes emphysema onset, pulmonary fibrosis and increases epithelial permeability. Upregulated in AE-COPD. Pre-clinical.	Upregulates adhesion molecules and leucocyte integrins. Endothelial injury and vascular dysfunction. Activates T-cells and is important for infarct healing	(9, 10)
TNF-α	Promotes upregulation of neutrophilic cytokines and MMPs. Upregulated in AE-COPD	Promotes immune cell extravasation	(7, 11, 12)
Targets which have undergone preclinical studies			
IL-8	Induces lung inflammation, promotes chemotaxis, mucus secretion and airway obstruction. Upregulated in AE-COPD	Promotes neutrophil and monocyte recruitment	(7, 8, 12)
IL-17	Pro-inflammatory, induces fibrosis, stimulates mast cells, promotes neutrophil recruitment, protease release and mucus secretion. Upregulated in AE-COPD	Promotes cardiomyocyte apoptosis and neutrophil recruitment	(13, 14)
IL-36	Pro-inflammatory, promotes neutrophil recruitment, mucus hypersecretion, emphysema, and small airway remodeling	Pro-inflammatory, promotes chemotaxis, endothelial and cardiomyocytes oxidative stress, cardiac fibrosis, and VCAM-1 upregulation	(15–17)
S100A8/9	Activates inflammasomes, induce release of pro-inflammatory mediator. Upregulated in AE-COPD	Activates inflammasomes, promotes IL-1β production, immune cell recruitment and apoptosis	(18–20)
TGF-β	Induces emphysema and airway remodeling. Promotes ECM secretion and proliferation of fibroblast and smooth muscle cells	Influences post-infarct remodeling	(7, 21)

### Neutrophils

In an AMI, neutrophil counts are found to be significantly raised as a result of mobilization from the bone marrow, and this is used as a predictor of adverse outcomes and mortality (25). Significant neutrophil recruitment occurs in which their phagocytic functions serve to contribute to the clearance of debris. However, the vast array of hydrolytic, oxidative, and pore-forming molecules released by an overabundance of phagocytic neutrophils, alongside their delayed removal from the infarct site, end up causing significant collateral heart tissue destruction (7, 26). Circulating neutrophil counts peak within 1–3 days and drop after day 4 (Figure 1). Neutrophils are recruited to the infarcted area within hours following myocardial IR injury. In 2016, Ma et al. further showed that neutrophils harvested from the heart on day 1 post-MI were N1 neutrophils (pro-inflammatory), that express highly pro-inflammatory mediators, whereas those isolated between days 5–7 were N2 (anti-inflammatory) neutrophils (8, 27). Strategies such as neutrophil CD11/CD18 inhibition have been effective in reducing MI size in animal models (28). Recent studies highlighted neutrophils as a source of S100A8/A9, and its timely pharmacological inhibition was shown to improve cardiac function (18, 29).

### Monocytes & macrophages

Following an AMI, monocytes from the bone marrow and spleen are mobilised into the circulation and drawn to the injury site by chemotaxis. Peripheral monocytosis serves as an indicator of potential adverse LV remodeling (30). Murine studies showed that pro-inflammatory classical, or Ly6C^high^ monocytes, peak in numbers between days 3 and 4. These monocytes then differentiate into pro-inflammatory M1 macrophages, responsible for producing proteases (MMPs), cytokines (IL-1, IL-6, TNF-α, IFN-γ), chemokines (CCL2), and growth factors, aiding in debris clearance and initiating wound healing (31). Subsequently, the recruitment of anti-inflammatory non-classical, or Ly6C^low^ monocytes, takes place, peaking around day seven (8, 24). As M1 macrophage numbers diminish, M2 anti-inflammatory macrophages take over, orchestrating wound healing in the subsequent reparative phase (Figure 1). However, a prolonged presence of M1 macrophages can intensify the pro-inflammatory phase, aggravate damage beyond the initially infarcted area, and lead to adverse LV remodeling. This underlines the potential cardioprotective benefits of pharmacologically inhibiting M1 macrophages (8, 32). Furthermore, monocytes can differentiate into antigen-presenting dendritic cells, initiating the adaptive immune system through the production of chemokines and cytokines (IFN-γ, IL-6), which in turn activate T cells (9, 33).

### Lymphocytes

Lymphocytes are key players in the cell-mediated cytotoxic innate immune response, with various subsets actively involved. In animal models, both T and B lymphocyte subsets have been observed infiltrating the injury site. Following an AMI, patients display an increase in pro-inflammatory CD4^+^ Th1 and cytotoxic CD8^+^ T cells and a reduction in anti-inflammatory and protective CD4^+^ Th2 cells. The prolonged presence of these detrimental lymphocytes is linked to poorer patient prognosis (34, 35). Moreover, an increase in peripheral B cells has been noted in AMI cases, with mature B cell infiltration peaking around day five. Zouggari et al., (36), demonstrated that B cells release CCL7, which mobilizes bone marrow Ly6C^high^ monocytes to the injury site, exacerbating tissue damage. They also demonstrated a potential therapeutic role following B cell genetic depletion, as evidenced by reduced systemic inflammation, LV remodeling and MI size (36). Additionally, natural killer (NK) cells and their receptor expressions are significantly reduced following an MI, with recent studies indicating a protective role for these cells in atherosclerosis (37).

### Platelets

Platelets are well-known for their critical role in thrombosis. Recently, they have also been identified as key players in inflammation, contributing to the innate immune response. They aggregate in injured areas, localising the inflammatory response and aiding in the formation of a provisional matrix. During inflammatory events like AMI or post-reperfusion, platelets release inflammatory mediators such as cytokines, chemokines, and platelet-derived growth factor (PDGF). Their pro-inflammatory activities include upregulating ICAM-1, VCAM-1, and selectins on leukocytes, triggering ROS production, activating macrophages and cytotoxic lymphocytes, and enhancing circulating microparticle production (38, 39). Studies also shows a link between innate immunity, platelet activation, and coagulation, known as immunothrombosis. Its abnormal activation in cardiovascular diseases increases the risk of cardiovascular events, including AMI (40). Extracellular DNA, a trigger for immunothrombosis in AMI, has been identified, with studies highlighting the potential of DNase I treatment to reduce infarct size in myocardial ischemia models (41). Moreover, platelets can influence leukocyte functions by forming platelet-leukocyte (P-L) aggregates through P-selectin/PSGL-1 interactions. An increase in P-L aggregates in peripheral blood has been noted post-AMI, suggesting their potential as early biomarkers for MI (42). Additionally, targeting these P-L aggregates with anti-platelet therapies has shown effectiveness in reducing inflammation and subsequent complications in MI patients.

## Inflammatory mechanisms in chronic obstructive pulmonary disease

Harmful particles and gases, notably in cigarette smoke, trigger inflammation and damage lung cells. Prolonged exposure leads to an abnormal and enhanced inflammatory response, hallmark features of COPD. This altered response contributes to tissue destruction (emphysema), increased mucus production (chronic bronchitis), and fibrosis (bronchiolitis) (43). Imbalances between oxidants and antioxidants, as well as proteases and antiproteases, are central to COPD development, exacerbating inflammation and lung tissue damage (44, 45). Even after the cessation of harmful exposure, the inflammatory process in COPD remains persistent and progressive, especially once symptomatic (46). This is attributed to airway remodeling, impaired macrophage clearance, oxidative stress, and hypoxia (47). Moreover, COPD is linked to a systemic inflammatory response, particularly notable in advanced stages or during AE-COPD, heightening the risk of associated comorbidities (48).

### Neutrophils

In COPD patients, high neutrophil counts correlate with poor outcomes and increased mortality (49, 50). However, these neutrophils exhibit compromised chemotaxis and release inflammatory mediators that contribute to alveolar damage (Figure 1) (51, 52). Both neutrophils and macrophages are key sources of MMPs, which are implicated in the breakdown of the extracellular matrix (ECM), airway obstruction, remodeling, and the development of emphysema (53, 54). MMP-12, in particular, is crucial for emphysema formation, as evidenced by MMP-12 deficient mice not developing emphysema post-cigarette smoke (CS) exposure (55). In COPD patients, MMP-2 and MMP-9 are potential biomarkers, with the highest expression levels observed in severe COPD cases (56). Specifically, the MMP-9 to tissue inhibitors of MMP-1 (TIMP-1) ratio has been linked to an increased mortality risk, indicating that targeting MMP-9 may help reduce this risk (57). Additionally, MMP-2 is implicated in ECM remodelling (58). Neutrophil elastase (NE), a neutrophil-derived serine protease, also plays a significant role in ECM degradation, influencing mucus secretion and fibroblast proliferation, which further contribute to airway obstruction and bronchial fibrosis (59, 60). Mice deficient in NE showed resistance to emphysema development in a CS exposure model (61). Moreover, excessive formation of neutrophil extracellular traps (NETs) has been associated with alveolar destruction (62).

### Monocytes & macrophages

In COPD, heightened macrophage recruitment within airways and lungs is a predictor of alveolar damage and disease progression (63). Pro-inflammatory M1 macrophages, prevalent in emphysematous regions, release a variety of inflammatory mediators. These mediators drive alveolar destruction via autocrine and paracrine pathways (Figure 1) (64–66). IL-1β and TNF-α intensify inflammation and lung tissue damage by upregulating neutrophilic cytokines and MMPs (Table 1) (5, 11). Mice lacking the IL-1 receptor exhibited resistance to emphysema and small airway remodeling in a CS exposure model (6). However, a clinical trial using an anti–IL-1R1 monoclonal antibody did not enhance lung function or quality of life in COPD patients (67). Recent studies have also delved into the role of the IL-36 sub-family in COPD (68, 69). IL-36 cytokines, primarily expressed in small airway cells, macrophages, and fibroblasts, are implicated in neutrophil recruitment, mucus hypersecretion, emphysema, and airway remodeling. Notably, COPD patients exhibit exacerbated symptoms due to a reduction in endogenous IL-36Ra in macrophages (15). IL-8, produced by macrophages, small airway cells, and endothelial cells, is also involved in mucus secretion and airway obstruction (70). Mice deficient in the IL-8 receptor, CXCR2, demonstrated protection against lung inflammation and DNA damage in a CS exposure model (71). IL-18 is notably elevated in the lungs of COPD patients, particularly in alveolar macrophages and epithelia, and its serum levels are higher compared to smokers and nonsmokers (72). This increase negatively correlates with pulmonary function and is linked to enhanced IFNγ production in severe COPD, contributing to remodeling responses (73). Similarly, IL-32 levels correlate positively with COPD severity, suggesting its potential as a biomarker for disease severity (74).

### Lymphocytes

COPD activates the adaptive immune system, characterised by the presence of B, T, and T-helper type 17 (Th17) cells, and a decrease in regulatory T-cells (Tregs) in the airways (Figure 1) (75). The progression of the disease sees the formation of lymphoid follicles, comprising B and T-cells, in the walls of small airways (76). Mice deficient in T and B-cells exhibited significantly reduced airway remodeling (77). A recent study noted an increased ratio of CD8^+^ to CD4^+^ cells in COPD patients, indicating a shift towards Th1/Tc1 dominance, which was associated with COPD exacerbation (78). Th17 cells, prominent producers of pro-inflammatory IL-17, are elevated in advanced COPD stages and contribute to CS-induced lymphoid neogenesis (13). Importantly, mice lacking the IL-17 receptor were protected from airway inflammation and fibrosis (14).

### Platelets

Platelets contribute to COPD-related inflammation through lung elastin breakdown and the formation of platelet aggregates (79). Elevated platelet counts and reactivity are associated with increased lung inflammation, reduced lung function, and greater disease severity in COPD patients (80). COPD is linked to a hypercoagulable state marked by raised coagulation factor levels, leading to a prothrombotic effect. This state features increased fibrinogen and coagulation factors, alongside decreased coagulation inhibitors. Systemic and neutrophilic inflammation contribute to these prothrombotic changes (81). While the exact role of immunothrombosis in COPD is still under investigation, it's believed to contribute to pathogenesis, potentially through NETs (82).

### Other inflammatory cells

In COPD, mast cells and eosinophils play significant roles in inflammation. Changes in mast cell populations in COPD patients include an increase in connective tissue mast cells (MC_TC_) and a decrease in mucosal mast cells (MC_T_), which are associated with airway remodeling and reduced lung function (83). The molecular mechanisms of mast cell involvement in COPD are still under investigation, but current hypotheses include their role in neutrophil recruitment, mucus hypersecretion through mast cell chymase, and tissue damage via enzymes like elastase, tryptase, and chymase (84). Eosinophil counts and related proteins may rise in certain COPD patients, particularly those with concurrent asthma (85, 86), although other studies report no significant increase or activation of eosinophils (86).

### Inflammatory mechanisms in acute exacerbations of chronic obstructive pulmonary disease

A hallmark of COPD is the occurrence of acute exacerbations, typically triggered by infections (bacterial or viral) or environmental factors (pollutants). These exacerbations are characterised by a sudden worsening of respiratory symptoms and an elevated inflammatory response. Consequences include aggravated mucus hypersecretion, bronchoconstriction, impaired gas exchange, respiratory muscle fatigue, hypoxemia, hypercapnia, respiratory acidosis, and compromised vascular perfusion. The frequency of AE-COPD is closely associated with declining lung function and increased mortality (87). Neutrophils predominantly mediate the inflammatory response in AE-COPD, although eosinophilia has also been linked to AE-COPD and its risk (88, 89). Elevated neutrophil counts have been documented in bronchoalveolar lavage fluid (BALF), bronchial walls, pulmonary microcirculation, and airways (90). During AE-COPD, various inflammatory mediators show increased levels compared to stable COPD, including serum CXCL8, e-selectin, TNF-α, IL-6, and more recently, MMP-9, IL-1β, IL-8, IL-17, IL-18, and S100A8/A9 (Table 1) (91–93). Serum IL-8 has proven more sensitive than TNF-α or IL-6 in predicting AE-COPD (12). Additionally, a clinical correlation has been established between increased NK cell presence and AE-COPD (94). In a mouse model of AE-COPD (CS exposure and viral infection), NK cells were found to produce more IFN-γ (95). Furthermore, evidence suggests that inflammatory mediators from the lungs can spillover into systemic circulation, causing chronic, non-resolving, low-grade systemic inflammation. This condition potentially leads to cardiovascular comorbidities, muscle weakness, and other systemic issues (21). The mechanism of this spillover is under debate. Currently, it is hypothesized to involve local activation of inflammatory pathways, elevating various inflammatory markers such as IL-6, IL-1β, TNF-α, CRP, and inflammatory cells in the peripheral blood (21, 96). Furthermore, the mobilization of inflammatory cells from the bone marrow, driven by factors like granulocyte-colony-stimulating factor (G-CSF), is believed to contribute to the increased presence of these cells in peripheral blood (97, 98). AE-COPD are also linked to an inflammation-driven prothrombotic state, featuring elevated platelet-monocyte aggregates, increased platelet count, endothelial activation, and heightened plasmatic prothrombotic markers (46).

## Inflammatory mechanisms in AMI and COPD

Over recent decades, a decline in the incidence and mortality of AMI, particularly ST-elevation myocardial infarction (STEMI), has been observed. Contrastingly, non-ST-elevation myocardial infarction (NSTEMI) cases are increasingly prevalent. Patients with NSTEMI often present with comorbidities or are of advanced age. Although significant focus has been placed on high-risk groups like diabetic patients, other equally at-risk populations, such as COPD patients, have received less attention (99). The prevalence of COPD in AMI populations, possibly underestimated due to general COPD underdiagnosis, ranges from 7% to 30% (43). The risk of AMI and its related mortality is markedly higher in COPD patients, especially soon after an AE-COPD (1). Cardiovascular diseases, predominantly AMI, account for around 50% of deaths in COPD cases. Notably, AMI is closely associated with COPD due to factors like systemic inflammation, thrombosis, and oxidative stress linked to COPD (43). The increased risk is also linked with the unique physiological crosstalk between the lungs and heart, which is unlike that in other organs. Indeed, research indicates that lung inflammation, as seen in conditions like SARS-CoV-2 infection, significantly impacts the heart due to the interconnectedness of the cardiopulmonary system (100). A substantial portion of the COPD disease burden relates to the management of cardiovascular comorbidities and infectious AE-COPD. AMI and COPD share common risk factors, including smoking, aging, and male sex, however the underlying mechanisms connecting them remain elusive. Systemic inflammation and hypoxia, stemming from COPD, are identified as independent risk factors for AMI (101). Despite extensive epidemiological research on both COPD and AMI, the pathophysiological link between them is less explored, due to the complexity of the multimorbidity involved.

**Figure 2:**
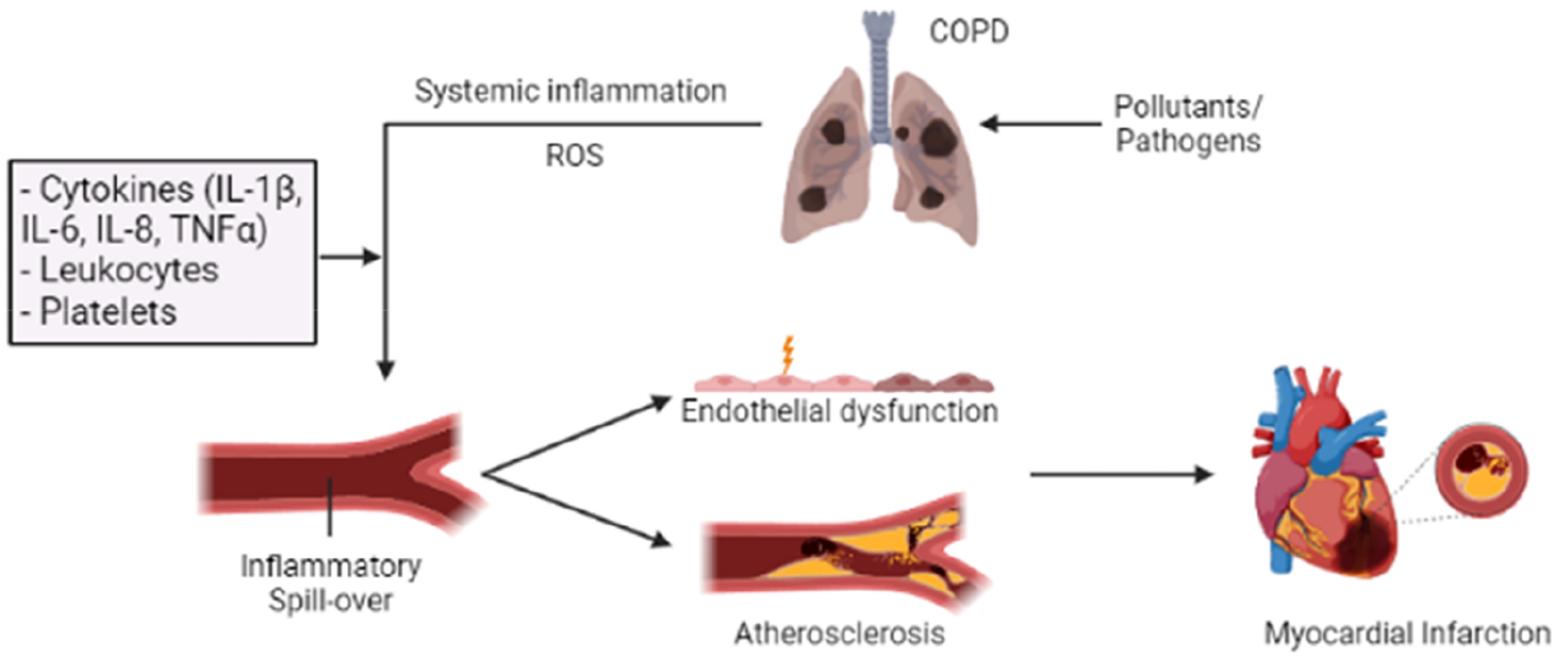
Overview of how inflammation in COPD can “spill over” into the systemic circulation, increasing the risk of AMI. Long-term exposure to pollutants and frequent acute exacerbations of chronic obstructive pulmonary disease (AE-COPD) can enhance and alter the inflammatory response in the lungs, resulting in a progressive and persistent inflammatory response. This COPD-related inflammatory response can promote systemic inflammation and oxidative stress through several inflammatory mediators (IL-1β, IL-6, IL-8, and TNF-α). Their actions can induce endothelial injury and vascular dysfunction through increased endothelial permeability and enhanced oxidative stress. Additionally, prolonged inflammation and oxidative stress can contribute to the development of atherosclerotic plaque and thrombosis, thereby increasing the risk of acute myocardial infarction (AMI). Patients with COPD exhibit a heightened platelet count and reactivity, ultimately increasing the risk of thrombotic events.

The inflammatory response in COPD can lead to endothelial damage and vascular dysfunction, though the underlying mechanisms warrant further investigation. Inflammatory mediators (such as IL-1, IL-6, IL-8, TNF-α, IL-36, and MMPs) released during COPD, can directly harm endothelial cells lining blood vessels, promoting atherosclerosis, the underlying cause of most AMI (102–104). Chronic lung inflammation promotes systemic inflammation and oxidative stress, which are key in atherosclerotic plaque formation (Figure 2). Prolonged inflammation and oxidative stress are known to elevate the risk of plaque development and rupture, leading to thrombosis and ischemic injury. COPD and cigarette smoke (CS) exposure further exacerbate this risk by increasing thrombosis through the secretion of procoagulant factors. COPD patients typically exhibit higher platelet counts and reactivity, along with increased coagulation factors, resulting in elevated thrombin levels and a greater risk of thrombotic events (103). A recent study focusing on the neutrophil-to-lymphocyte ratio (NLR), an indicator of systemic inflammation, found that a high NLR in COPD patients is associated with an increased risk of AMI, stroke, and mortality, suggesting systemic inflammation's role in cardiovascular disease development within this group (105). Moreover, elevated troponin levels, particularly during AE-COPD and linked to increased neutrophil recruitment, have been noted in COPD patients (106). Patel et al. demonstrated that frequent AE-COPD result in increased arterial stiffness and elevated troponin and BNP levels, correlating these disturbances with the inflammatory response marked by raised IL-6 and IL-8 during AE-COPD (107).

The survival rate of patients with COPD who are subject to an AMI is significantly worse than non-COPD patients. This increased vulnerability to myocardial injury in COPD patients is multifactorial, attributed to airway remodeling driven by inflammation-induced vascular changes and deteriorating lung function. Notably, pulmonary artery intima thickening has been observed not only in mild to moderate COPD patients but also in smokers, indicating that such vascular alterations can manifest early in disease progression (108). Furthermore, pulmonary hypertension (PH), a common complication of severe COPD, significantly contributes to AMI and heart failure. COPD leads to PH through sustained hypoxia, hypoxic vasoconstriction, inflammation in the lungs and systemic inflammation, as well as endothelial dysfunction. These factors result in pulmonary arteriole remodeling and narrowing, exacerbating hypoxia, and elevating pulmonary blood pressure (109). Hypoxia heightens ROS production and oxidative stress, impacting vascular function, cardiac output, and blood pressure. Recent studies have highlighted the role of hypoxia-inducible factor (HIF)-α in promoting vascular remodeling and atherosclerosis via inflammatory pathways (110). Current research underscores the inflammatory response as a pivotal element in the COPD-AMI connection, suggesting that targeting inflammation could be a promising approach in treating cardiovascular disease among COPD patients.

## Therapeutic strategies for targeting inflammation in AMI and COPD

A potential therapeutic target for limiting COPD progression, infarct size and adverse remodeling while enhancing prognosis could be to target the pro-inflammatory responses observed during COPD and myocardial IR injury (8, 111, 112). The current standard anti-inflammatory therapy for COPD is corticosteroids, often combined with a long-acting bronchodilator to enhance lung function and reduce exacerbations. The impact of corticosteroids on AMI incidence and outcomes in both COPD and non-COPD patients is subject to conflicting findings. Inhaled corticosteroids may potentially reduce AMI risk in COPD patients, although some studies show no significant effect (113, 114). In contrast, oral corticosteroid use increases AMI risk in COPD patients (115). In non-COPD patients, corticosteroid use for AMI treatment raises concerns about adverse effects like impaired wound healing and remodelling (116). The role of corticosteroids in AMI treatment remains a topic of research and clinical debate, necessitating further investigation into their potential benefits and risks. Aspirin, a standard therapy for AMI patients to prevent blood clots and reduce the risk of new MI or stroke, may also provide pulmonary benefits and decelerate COPD progression (117). A recent trail suggests its potential in reducing cardiovascular complications in COPD patients, warranting further investigation (118).

Several other studies and clinical trials have focused on individual components of the inflammatory adhesion cascade, showing some benefits for COPD and AMI independently (Table 1). However, elevated levels of inflammatory mediators in these diseases do not necessarily suggest that their inhibition will be an effective anti-inflammatory strategy. For instance, while TNFα levels are high in both AMI and COPD, inhibiting it has not demonstrated clinical benefits in either of these conditions (119). The pleiotropic effects of inflammatory cytokines on the immune system make them attractive targets for reducing inflammation. Several clinical trials have been undertaken to assess therapies targeting key inflammatory cytokines, especially IL-1, IL-6, and TNF-α (Table 1). However, there has been a lack of studies examining the impact of anti-inflammatory treatments in animal models or patients with both COPD and AMI. Therefore, this review will focus only on targets that have demonstrated benefits in both COPD and AMI independently, recognizing that potential dual therapy targets warrant further research.

### Therapeutic targets in the interleukin-1 super family

The IL-1 superfamily (IL-1F), encompassing eleven cytokines, plays a pivotal role in modulating the innate immune response. This family includes both pro-inflammatory and anti-inflammatory cytokines that influence integrin expression on target cells and stimulate cytokine release from stromal cells. The pro-inflammatory members are IL-1α, IL-1β, IL-18, IL-36α, IL-36β, IL-36γ, and IL-33. The anti-inflammatory cytokines are divided into immunosuppressive (IL-37 and IL-38) and antagonist cytokines (IL-1Ra, which antagonizes IL-1α and IL-1β, and IL-36Ra, targeting IL-36α, IL-36β, and IL-36γ). Except for IL-1Ra, all members of the IL-1 family are initially produced as precursor proteins, requiring proteolytic cleavage to become active, mature proteins (120). IL-1 cytokines (IL-1α and IL-1β) have garnered significant attention; these agonists, located in the cytoplasm, are expressed in various cells, including neutrophils, monocytes, and macrophages. However, some members, like the IL-36 cytokines, have been recently discovered, and research on their functions remains limited (121). IL-1F cytokines are often the earliest and most upstream responders to injury, making them promising targets for managing inflammatory disorders (122). Given their critical role in mediating inflammation, IL-1F members could be key to understanding and addressing myocardial and pulmonary disturbances.

### Targeting IL-1 in AMI and COPD

Experimental models that target IL-1 in myocardial IR injury have shown promise. Toldo et al. (123) reported that anakinra, a recombinant human IL-1 receptor antagonist (IL-1Ra), significantly reduced infarct size and improved LV ejection fraction in mice following myocardial IR injury (123). Likewise, a study in rat hearts indicated that IL-1Ra overexpression protected against IR injury by attenuating cell death and reducing infarct size through its anti-inflammatory properties on neutrophils (124). Clinical studies using anakinra or canakinumab, an IL-1β monoclonal antibody, in STEMI or NSTEMI patients have been mixed. Morton et al. (125) conducted a double-blinded randomized phase II trial with anakinra in NSTEMI patients, finding decreased CRP levels but increased adverse outcomes (recurrent MI, stroke, and death) over one year (8, 125). Conversely, the CANTOS trial by Ridker et al. (126) demonstrated that canakinumab significantly reduced nonfatal MI, stroke, or cardiovascular death in high-risk patients over 3.7 years (126). This was the first major clinical trial to show that targeting inflammation can confer modest cardiovascular benefits in very high-risk patients and has been credited with providing an exciting glimpse at the potential for using anti-inflammatory therapies for treating cardiovascular diseases.

In CS exposure models, IL-1 inhibition also showed effectiveness. Castro et al. (127) found that an anti-IL-1β antibody significantly reduced CS-induced lung inflammation (127). Churg et al. (6) reported protection against emphysema and airway remodeling in IL-1R knockout mice exposed to CS (6). A phase I clinical study by Hernandez et al. (128) using anakinra in healthy volunteers exposed to inhaled lipopolysaccharide (LPS) showed reduced airway neutrophilia and cytokine levels (IL-1β, IL-6, and IL-8) (128). In a phase II trial, Calverley et al. (67) tested MEDI8968, an IL-1R1 monoclonal antibody, in moderate-to-severe COPD patients, finding initial inflammation reduction but no significant changes in AE-COPD rate, lung function, or quality of life compared to placebo (67). Overall, this trial suggested potential benefits for COPD patients with high neutrophil counts, though current serum biomarkers might not adequately identify these patients.

### Targeting IL-36 in AMI and COPD

IL-36, a relatively recent member of the IL-1 family discovered about two decades ago, plays a key role in pro-inflammatory mediator production, immune cell activation, and antigen presentation (129). As well as amplifying IL-1 effects, IL-36 is also a mediator of inflammation in its own right. Indeed, its critical role in psoriasis, equalling if not surpassing that of IL-1, is well established with emerging roles in Crohn disease and rheumatoid arthritis recently identified (130–132). Research into IL-36 as a pro-inflammatory cytokine in various inflammatory diseases is extensive, but studies in AMI or COPD are more recent.

In experimental models of myocardial IR injury, inhibiting IL-36R has shown success. In 2019, Wei et al. found that administering recombinant mouse IL-38 in mice post-IR injury reduced inflammation, cardiac fibrosis, and myocardial injury, suggesting IL-38 could benefit cardiac remodeling after MI (133). More recently, we demonstrated that treatment with IL-36Ra in mice with myocardial IR injury significantly reduced infarct size and neutrophil recruitment, and improved blood flow. The mechanism involved attenuating endothelial and cardiac myocyte oxidative damage and VCAM-1 expression (16, 134). In the context of COPD, while the IL-36/IL36R pathway's pro-inflammatory role in the lungs is well recognized, its protective potential is still emerging. Baker et al. (15) reported reduced virally induced IL-36-mediated inflammation in COPD using IL-36Ra (Spesolimab) or an IL-36R blocking antibody. This was mechanistically explained by blocking the crosstalk between virally stimulated COPD small airway epithelial cells and small airway fibroblasts (15). Additionally, their 2021 study showed that IL-36R knockout mice had diminished lung inflammation and neutrophil recruitment in a CS exposure model (17). These findings suggest a novel role for IL-36 in both myocardial IR injury and COPD and necessitate further exploration in a comorbidity model.

### Therapeutic targets beyond IL-1F members

#### Targeting interleukin-6

IL-6, released from immune cells after myocardial IR injury and during COPD, exhibits both anti- and pro-inflammatory effects, notably as a potent inducer of CRP secretion. Vanfleteren et al. (135) identified elevated IL-6 plasma levels exclusively in COPD patients with cardiovascular comorbidities (135). AMI studies in mice with IL-6 genetic depletion or pharmacological inhibition show mixed outcomes on myocardial infarction size and LV remodeling, with some studies indicating worsened outcomes post-inhibition (136–138). Broch et al. (139) reported a phase II trial testing tocilizumab, an IL-6R monoclonal antibody, in STEMI patients, finding increased myocardial salvage and decreased microvascular obstruction, although infarct size was unchanged after 7 days (139). Inhibition of IL-6 also demonstrated to be beneficial in COPD. Indeed, Wei et al. (10) isolated human and mouse primary bronchial epithelial cells from COPD patients and mice. They showed IL-6 neutralizing antibody (IL-6 Ab) to attenuate airway mucus hypersecretion in COPD (10).

#### Targeting S100A8/A9

S100A8/A9, or calprotectin, part of the S100 DAMP family, is a prominent neutrophil and monocyte protein secreted during inflammation and infection. It plays a role in pro-inflammatory mediator production but can also exhibit anti-inflammatory effects under specific conditions. In myocardial IR injury models, inhibition of S100A8/A9 has been effective. Marinković et al. (29) observed reduced inflammatory cell recruitment and improved cardiac function following short term treatment with ABR-238901, an S100A8/A9 inhibitor (29). Similar improvements in cardiac function were seen in a study of mice transplanted with bone marrow from S100A9 knockout mice (18). However, prolonged ABR-238901 treatment worsened cardiac function and accelerated LV remodeling (140). These findings underline the need for timely interventions. Clinically, S100A8/A9 levels have been linked with recurrence risk and poorer prognosis (141). Despite recent increases in our understanding of how S100A8/A9 is involved in several lung diseases, we are still at an early stage in our current understanding of its potential protective role in COPD. Zhao et al. (20) found that ABR-238901 prevented LPS-induced lung injury in mice, primarily through NLRP3 pathway suppression (20). Conversely, recombinant S100A8/A9 reduced acute lung injury in LPS-challenged mice in another study (142). These varied outcomes, potentially influenced by differences in mouse species, treatment timing, and experimental setups, may underscore the dual pro- and anti-inflammatory nature of S100A8/A9. Additionally, S100A8 was found to protect human alveolar cells against emphysema and injury (143).

## Optimizing anti-inflammatory therapies for AMI and COPD

While various components of inflammatory process have shown promise in reducing infarct size and slowing COPD progression, their efficacy in a comorbidity context involving AMI in COPD patients remains untested. Consequently, the applicability of these strategies to such patients is uncertain. Additionally, several potential targets not covered in this review failed in clinical translation, possibly due to discrepancies in experimental design, therapeutic intervention timing, dosing, and the distinct pathophysiological mechanisms in animal models vs. humans. Experimental models often do not fully mirror human pathogenesis, especially considering that most COPD patients have comorbidities like atherosclerosis and AMI, are typically older, and frequently use multiple medications. Many animal studies also neglect sex differences in outcomes (8).

Non-resolving inflammation, which fails to naturally subside, plays a pivotal role in the pathophysiology of both COPD and AMI. In AMI, it contributes to adverse remodeling and advanced heart failure, leading to a poor prognosis, while in COPD, it triggers systemic effects, contributing to comorbidities like cardiovascular diseases, including AMI (21, 144). The timing of therapeutic intervention significantly influences inflammation resolution. Clinical trials administering anti-inflammatories days or months after AMI aim to target resolution but often miss the initial inflammatory peak, resulting in limited cardiovascular benefits (126). Recent research explored the benefits of very early anti-inflammatory therapy in AMI patients, showcasing its potential for salvaging myocardial tissue (139). Nevertheless, tackling persistent inflammation remains challenging, potentially necessitating sustained or additional therapeutic approaches. Similarly, in COPD patients, commonly used anti-inflammatories like corticosteroids have exhibited limitations in modulating non-resolving inflammation, even after smoking cessation (145). Unfortunately, no highly effective anti-inflammatory treatment successfully targets persistent inflammation. Therefore, understanding the molecular mechanisms governing resolution onset is imperative, offering the potential for developing resolution-based strategies, expanding research horizons, and potential therapeutic breakthroughs.

Enhancing clinical success may hinge on patient stratification and selection, particularly in COPD, a heterogeneous disease with various distinct phenotypes beyond genetic factors. This diversity also applies to AMI. COPD progression varies widely among patients, and factors like the degree of airway obstruction, pulmonary emphysema, chronic bronchitis, and the frequency of acute exacerbations do not fully capture this heterogeneity. Understanding clinical phenotypes and linking them to underlying pathophysiological mechanisms via inflammatory biomarkers is crucial. This approach allows for targeted therapy against specific anti-inflammatory markers predominantly expressed in individual patients. Moreover, developing new anti-inflammatory targets necessitates testing in animal models that more accurately represent clinical scenarios and through meticulous patient selection. Pursuing an inflammatory target that optimizes perfusion in pulmonary and coronary microcirculation, minimizes damage, and enhances patient prognosis remains a valuable endeavour.

## Concluding remarks

Over recent decades, substantial progress has been achieved in understanding the pathophysiological intricacies of COPD and AMI, with a particular emphasis on the pivotal role of inflammation as a connecting thread between these conditions. Despite these advances, the understanding of inflammatory interactions in a comorbidity context remains elusive. Furthermore, while specific inflammatory targets have been identified for therapeutic intervention, their efficacy in treating concurrent COPD and AMI remains unclear. This gap underscores an essential direction for future research, particularly given the variability in clinical manifestations and patient responses. Looking ahead, the adoption of personalized medicine, finely tuned to the unique inflammatory profiles of individuals, promises to yield more effective and precise treatment modalities. Hence, a deeper and more nuanced comprehension of the shared inflammatory pathways in COPD and AMI is imperative to enhance patient care and clinical outcomes.
